# Calibration-Free Roadside BEV Perception with V2X-Enabled Vehicle Position Assistance

**DOI:** 10.3390/s25133919

**Published:** 2025-06-24

**Authors:** Wei Zhang, Yilin Gao, Zhiyuan Jiang, Ruiqing Mao, Sheng Zhou

**Affiliations:** 1 Information and Communication Engineering, Shanghai University, Shanghai 200444, China; wei_zh@shu.edu.cn (W.Z.); gaoyilin@shu.edu.cn (Y.G.); 2 Electronic Engineering, Tsinghua University, Beijing 100190, China; mrq20@mails.tsinghua.edu.cn (R.M.); sheng.zhou@tsinghua.edu.cn (S.Z.)

**Keywords:** cellular-V2X (C-V2X), roadside bird’s eye view (BEV) perception, calibration-free perception

## Abstract

Roadside bird’s eye view (BEV) perception can enhance the comprehensive environmental awareness required for autonomous driving systems. Current approaches typically concentrate on BEV perception from the perspective of the vehicle, requiring precise camera calibration or depth estimation, leading to potential inaccuracies. We introduce a calibration-free roadside BEV perception architecture, which utilizes elevated roadside cameras in conjunction with the vehicle position transmitted via cellular vehicle-to-everything (C-V2X) independently of camera calibration parameters. To enhance robustness against practical issues such as V2X communication delay, packet loss, and positioning noise, we simulate real-world uncertainties by injecting random noise into the coordinate input and varying the proportion of vehicles providing location data. Experiments on the DAIR-V2X dataset demonstrate that the architecture achieves superior performance compared to calibration-based and calibration-free baselines, highlighting its effectiveness in roadside BEV perception.

## 1. Introduction

**Figure 1:**
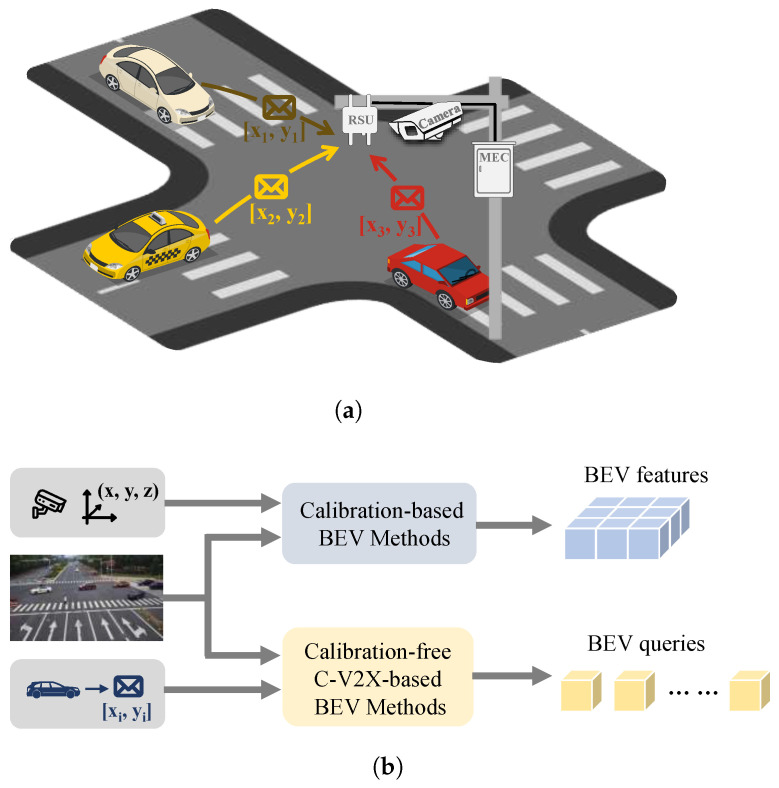
C-V2X enables cooperative perception between vehicle-side and roadside agents, where vehicle positions are transmitted to assist roadside BEV perception, ensuring accurate perspective transformation without reliance on camera calibration in calibration-based methods. (**a**) Description of vehicles transmit positions to the roadside unit (RSU) via C-V2X. (**b**) Description of calibration-based and calibration-free C-V2X-based BEV methods.

Bird’s eye view (BEV) perception is critical for intelligent transportation systems (ITSs), as it offers a comprehensive view of surroundings. Existing BEV perception methods that rely on images primarily target vehicles. Early BEV techniques, such as inverse perspective mapping (IPM) [1], utilize homography-driven transformations to convert perspective images onto a two-dimensional plane. Despite their computational efficiency, these methods face challenges in urban settings with irregular terrain and mobile objects. Multi-layer perceptron (MLP)-based approaches [2,3,4] attempt to directly learn projections from perspective views (PVs) to BEV but struggle with issues related to depth perception and spatial coherence. Transformers [5,6,7] employ attention mechanisms to capture extensive dependencies across multiple views, addressing limitations found in convolutional neural network (CNNs) and MLPs. The precision of camera intrinsics, extrinsics, and depth assessments is vital to these methodologies, as depicted in Figure 1b. Nonetheless, calibration inaccuracies can substantially diminish perceptual accuracy.

The perception of roadside BEVs is improved by the combination of elevated cameras and mobile edge computing (MEC), which enhances computational capabilities and environmental awareness. Perception data can be transmitted to vehicles to facilitate driving assistance through the use of cellular vehicle-to-everything (C-V2X) [8]. However, the calibration of roadside cameras poses a challenge due to the potential for inaccurate perception outcomes as a result of errors in intrinsic and extrinsic parameters. In addition, the calibration of sensors on roadside infrastructure can vary significantly due to environmental changes. This makes static calibration very challenging. Therefore, a method that can achieve cooperative perception without depending on accurate calibration is very meaningful. Calibration-free methods, such as PYVA [9] and CBR [10], have been developed to address this issue. Nevertheless, these solutions are limited by their low detection accuracy in complex scenarios with substantial occlusions. Consequently, their practical application is limited.

In order to address the current challenges, we suggest the implementation of calibration-free roadside BEV perception with V2X-based vehicle position assistance. This method proposes that vehicle-side data, such as vehicle positions, can improve roadside BEV perception through C-V2X, as illustrated in Figure 1a. In our assumed deployment scenario, each roadside unit (RSU) is equipped with a monocular camera to capture visual information. Vehicles located within approximately 100 m of the RSU can transmit their real-time positions to the RSU via the C-V2X communication protocol. These positions are then used to assist the RSU in image-based BEV detection tasks. However, V2X communication may suffer from packet loss, multi-path interference, or latency, leading to an instability of received positions [11]. In addition, the penetration rate of C-V2X-equipped vehicles is often incomplete, meaning position information is only partially available in many scenes. To remain robust in the face of V2X uncertainty and limited participation, we vary the percentage of vehicles that transmit location data and inject noise into the received data during training and inference. The positions are transformed into reference points that are in alignment with the image space as a result of the collaboration between roadside image features and vehicle positions, which enables precise perspective transformation. We incorporate random noise into the input positions to improve the model’s generalization abilities and minimize the domain gap between vehicle positions and image features.

Our contributions are summarized as follows:

- We introduce a roadside calibration-free BEV perception framework that utilizes C-V2X-transmitted vehicle positions to improve perception. This approach eliminates the dependence on camera calibration metrics and reduces performance drops caused by calibration inaccuracies.
- To simulate real-world communication imperfections such as V2X latency and limited participation, we inject uniform coordinate noise and vary the availability of position input across vehicles. This enhances the robustness of our model under practical constraints and promotes better generalization between coordinate and image feature spaces.
- Experimental results on the DAIR-V2X dataset showcase the method’s excellent performance compared to calibration-based and calibration-free baselines in roadside BEV perception tasks. The proposed design remains effective even when only a portion of vehicles provide position data, proving its adaptability to limited V2X penetration scenarios.

## 2. Related Works

### 2.1. Image-Based BEV Perception

Early approaches to BEV perception primarily relied on geometry-based methods. Inverse perspective mapping (IPM) [1] and stereo-based homography projection [12,13] use camera calibration parameters to project perspective view (PV) images into a BEV plane. While efficient, these methods were sensitive to ground assumption violations and performed poorly in complex urban environments. To overcome these limitations, learning-based methods were introduced. MLP-based methods, such as VED [3], VPN [4], and PON [14], attempted to learn the mapping from PV to BEV directly. However, these methods often lacked spatial precision and struggled with occlusion or depth ambiguity. HDMapNet [15] addressed spatial misalignment by employing bidirectional projection, yet its reliance on image-only inputs limited its adaptability. Recent transformer-based frameworks have significantly improved BEV perception. BEVFormer [5] and BEVDepth [16] utilize spatial–temporal attention and depth-supervised geometry priors, achieving strong performance under calibrated multi-view settings. BEVHeight [17] further introduces height priors into BEV construction, which improves vertical object structure modeling. CoBEV [18] utilizes height maps and depth features from monocular roadside views to improve 3D object detection, which enhances detection accuracy in complex road scenarios. Nevertheless, these methods depend heavily on accurate camera calibration or depth prediction, making them vulnerable to real-world deployment errors.

### 2.2. V2X-Assisted BEV Perception

To address the limitations caused by calibration dependency and limited perception range, cooperative BEV perception via V2X has emerged. CoBEVT [19] leverages multi-agent information fusion using cross-attention between agents’ BEV features, improving robustness in multi-view settings. V2X-ViT [20] further adopts vision transformers with early fusion, achieving strong collaboration by modeling inter-agent dependencies. FedBEVT [21] proposes federated multi-agent training, emphasizing privacy-preserving fusion while preserving performance. These works focus on vehicle-side collaboration and require multi-agent BEV features or synchronized fusion at the model level. However, most current works assume full V2X coverage and perfect position alignment. In contrast, our method is designed for partial V2X environments and does not require explicit BEV feature fusion from vehicles. Instead, it injects position noise and random availability to simulate real-world imperfections, achieving robust and calibration-free BEV detection under infrastructure-side settings.

## 3. Algorithm Framework

**Figure 2:**
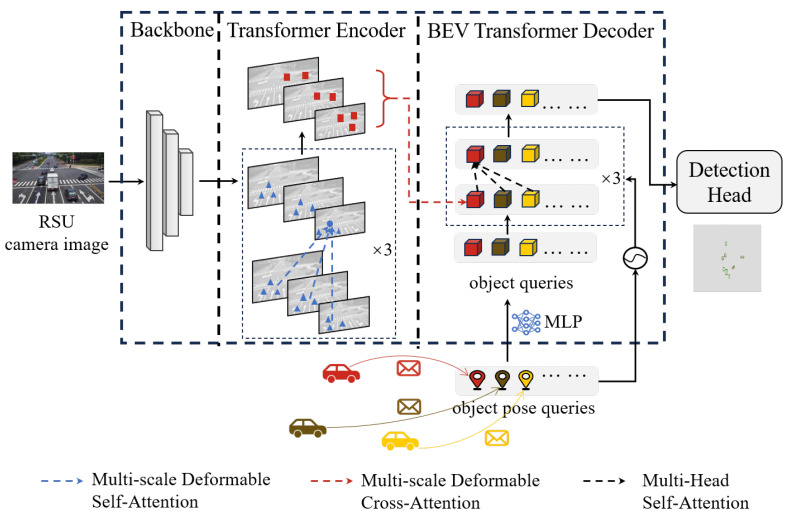
Overview of the proposed roadside BEV perception framework consisting of the backbone, transformer encoder, BEV transformer decoder, and detection head. The roadside camera captures images, while the vehicle’s 2D positions, transmitted via C-V2X, are incorporated to assist feature extraction and alignment. The deformable cross-attention module maps 2D position-derived reference points to the feature space, enabling accurate BEV perception without relying on camera calibration.

To achieve calibration-free BEV perception at the roadside, our method combined infrastructure-side visual features with vehicle-side position information transmitted through C-V2X. The overall framework was designed to extract spatial features from monocular images and align them with vehicle-provided coordinates for accurate BEV detection. As illustrated in Figure 2, the architecture consists of three core modules. The backbone network extracted multi-scale features from the input image. The encoder was used to improve these extracted features. Finally, the decoder transformed received vehicle positions into object queries to perform detection in the BEV space.

### 3.1. Transformer Encoder

The ResNet [22] backbone combined with the feature pyramid network (FPN) [23] was used to extract multi-scale feature maps at four pyramid levels, denoted as $F_{l}\in R^{C\times H_{l}\times W_{l}}$, where l∈{1, 2, 3, 4}, *C* is the number of channels, and $H_{l},W_{l}$ represent the height and width of the feature map at level *l*. Each $F_{l}$ was first projected to a unified embedding space with 256 channels via a 1 × 1 convolution. Then, the resulting feature map was reshaped into a sequence of flattened tokens with shape $H_{l}W_{l}\times 256$. The multi-scale token sequences, denoted as $F_{l}^{\prime }\in R^{H_{l}W_{l}\times 256}$, were fed into the deformable attention-based transformer encoder for further processing. This design ensures that spatial information is preserved while enabling efficient multi-scale attention computation.

As suggested in the deformable DETR [24], we used the multi-scale deformable self-attention module to improve the features. In contrast to traditional attention techniques, which require dense pairwise interactions, this module minimizes processing costs by focusing on a limited number of reference points that surround each query. Deformable self-attention effectively captures local spatial information by selectively attending to important locations, allowing for efficient multi-scale feature fusion while preserving computing efficiency.

### 3.2. BEV Transformer Decoder

**Figure 3:**
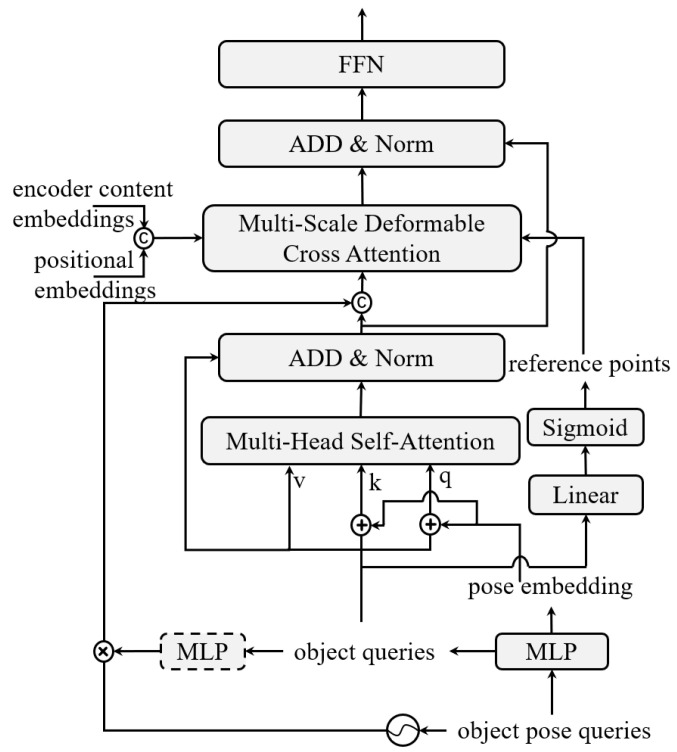
Illustration of the BEV transformer decoder.

The multi-head self-attention module and the multi-scale deformable cross-attention module were the two primary components of the decoder, as illustrated in Figure 3.

To simulate the transmission latency and localization error, the vehicle positions received through C-V2X were perturbed by adding a random offset within the range of [−10, 10] meters. This range is supported by prior studies showing that typical GPS errors in vehicle communication scenarios range from 1 to 10 m due to factors such as multipath propagation and receiver inaccuracies [25]. Therefore, our training setup realistically reflects the upper bound of C-V2X positioning errors, particularly under urban or obstructed environments. The perturbed results were subsequently normalized and converted into a 2D coordinate matrix in the following manner:

(1) $$p_{\mathrm{obj}}=\frac{p+e}{p_{\max}},\;e∼\mathrm{Uniform}\left(-10\;m,10\;m\right),$$

where p denotes the original positions, e represents the random offset, and $p_{\max}$ is the normalization scale factor. The object queries $(q_{\mathrm{obj}}$) and the pose embedding are generated by passing these normalized 2D positions through MLP.

By collecting dependencies across various elements in the queries, the multi-head self-attention module was implemented to enhance the relationships between object queries. This improves the contextual representation of each query in the decoder.

By using a sigmoid activation function and a linear transformation, the object queries were used to generate reference points ($p_{\mathrm{ref}}$) while maintaining the spatial relationships of the objects. Starting with the second decoder layer, the decoder integrated positional encoding, combining coordinate information with both the object queries and multi-scale encoder features by concatenating to further improve spatial alignment. This approach is inspired by conditional DETR [26]. The decoder can concentrate on relevant regions and produce accurate detection results because of this technique, which directly embeds spatial relationships into the feature space. Features from various scales are combined in the multi-scale deformable cross-attention module, which is defined as follows:

(2) $$\begin{array}{c} \mathrm{MSDeformAttn}\left(q,p_{ref},{\left\{F_{l}^{\prime }\right\}}_{l=1}^{L}\right)=\sum_{m=1}^{M}W_{m}\sum_{l=1}^{L}\sum_{k=1}^{K}A_{mlk}\cdot W_{m}^{\prime }F_{l}^{\prime }\left(p_{ref}+\Delta p_{mlk}\right) \end{array}$$

where $\Delta p_{mlk}$ denotes the sampling offsets and $A_{mlk}$ is the attention weight for the *k*-th sampling point on the *l*-th feature level and *m*-th attention head. The weight matrices $W_{m}$ and $W_{m}^{\prime }$ are learnable. The cross-attention module predicts the attention weights $A_{mlk}$ and offsets $\Delta p_{mlk}$.

The decoder converted vehicle positions into a meaningful representation that facilitated the extraction of BEV features. Unlike traditional BEV detection methods that initialize a fixed grid of BEV queries to span the entire spatial map, our approach directly utilizes vehicle-derived 2D positions (object pose queries) as dynamic inputs to the decoder. These pose-based queries are transformed from real-world C-V2X-transmitted coordinates and carry rich spatial priors corresponding to real object locations. As such, they act as semantic anchors in BEV space, enabling the decoder to focus attention on informative regions without requiring dense BEV queries. Its components work together to guarantee accurate class and bounding-box regression and robust feature alignment, effectively adapting to diverse and unstable input scenarios.

### 3.3. Training Target

Our loss function used the deformable DETR design [24] and the Hungarian algorithm [27] for bipartite prediction–ground truth matching. Additionally, we added 3D object detection to this framework. We used focused loss [28] for classification, weighting it at two to emphasize its importance. We used seven factors to forecast three-dimensional bounding boxes for box regression, namely orientation θ, dimensions (w,h,l), and center positions (x,y,z). The regression loss combines L1 and GIoU losses [29]. To align 3D boxes in the BEV plane, GIoU was applied to the BEV projection. The total loss is:

(3) $$L=\lambda_{\mathrm{cls}}L_{\mathrm{cls}}+\lambda_{L1}L_{L1}+\lambda_{\mathrm{GIoU}}L_{\mathrm{GIoU}},$$

where $L_{\mathrm{cls}}$ is the focal classification loss, $L_{L1}$ is the L1 regression loss, and $L_{\mathrm{GIoU}}$ is the GIoU loss for BEV bounding boxes. The classification, L1 regression, and GIoU contributions are balanced by the weights $\lambda_{\mathrm{cls}}$, $\lambda_{L1}$, and $\lambda_{\mathrm{GIou}}$. In our experiment, $\lambda_{\mathrm{cls}}$, $\lambda_{L1}$, and $\lambda_{\mathrm{GIou}}$ are set as 2, 5 and 2, respectively.

## 4. Experiments

### 4.1. Datasets

Our experiments were conducted on the DAIR-V2X and DAIR-V2X-I datasets [30], providing real-world cooperative perception data on a large scale. The DAIR-V2X dataset contains 12,424 images that were acquired from infrastructure-mounted cameras. These images were divided into 8800 frames for training and 3624 frames for validation. Similarly, DAIR-V2X-I contains approximately 10,084 frames, which were divided into training, validation, and assessment sets at a ratio of 50%, 20%, and 30%, respectively. All evaluations were conducted on their respective validation sets as in [10,17].

To guarantee comparability, we specifically used roadside camera data from both datasets in this study. Following the KITTI benchmark [31], we divided the annotated objects into three difficulty levels, which were easy, moderate, and hard. The division was based on object occlusion, truncation, and 2D bounding box height in the image. Specifically, easy samples are fully visible with minimal truncation and large bounding box sizes (the height is greater than 40); moderate samples have partial occlusion and slightly smaller bounding boxes (the height is between 40 and 25); hard samples are heavily occluded, truncated, or appear with small bounding boxes (the height is smaller than 25). This classification provides a granular understanding of detection robustness in varying real-world traffic scenarios and is widely adopted in 3D detection benchmarks to facilitate fair and informative performance comparisons.

### 4.2. Implementation Details

Baselines: We established our baseline by randomly picking the proportion of vehicles transmitting vehicle positions via C-V2X from {25%, 50%, 75%, 100%} during training. This models C-V2X penetration uncertainty such as in early-stage deployments where only a subset of vehicles is equipped with V2X capabilities and asynchronous communication, thereby improving its generalization capabilities in real-world scenarios.

To guarantee independent comparisons, we aligned our baseline experiments with the configurations outlined in CBR [10]. CBR introduces Gaussian noise to the rotation angles for calibration-based methods, such as ImVoxelNet [32], to simulate calibration inaccuracies. This is modeled as

(4) $$\theta_{n}=x_{n}\cdot n_{\mathrm{range}}$$

where $x_{n}∼N\left(\mu ,\sigma^{2}\right)$, with μ=0 and $\sigma =\frac{1}{3}$. Noise levels $n_{\mathrm{range}}$ are chosen from the range {0.1, 0.2, 0.5, 1.0} to assess robustness in the presence of varying calibration errors. In the context of calibration-free methods, we contrasted our methodology with that of CBR.

Ablation Studies: We conducted an ablation study to evaluate the effectiveness of adding vehicle positions. The experiment compared our strategy to a scenario where no vehicles communicated vehicle positions (0%). This let us assess C-V2X-based cooperative perception and the model’s ability to adjust without external positional information. To investigate the effect of coordinate noise injection on generalization, we designed a controlled ablation experiment before and after applying coordinate noise during training.

Experiment Parameters: The perception range was set to [90 m,90 m,5 m] following CBR, and the input images were resized to 512 × 512. The Adam optimizer was implemented, and the learning rate was established at $10^{-4}$. Training was conducted on two NVIDIA GeForce RTX 4090 GPUs with a batch size of 32 per GPU over 600 epochs.

### 4.3. Experimental Results

#### 4.3.1. Comparison with Baselines on DAIR-V2X

Table 1 presents a comparison of our method with calibration-based and calibration-free approaches utilizing the DAIR-V2X dataset. The evaluation metrics consisted of $AP_{3D}$ and $AP_{BEV}$ for the car category at IoU = 0.5, employing the R40 metric. The results indicate that ImVoxelNet’s performance decreases rapidly with increased calibration noise, decreasing from 47.6% to 19.7% $AP_{3D}$ for easy samples. This highlights its significant dependence on accurate camera calibration. Our method exceeds ImVoxelNet in the presence of calibration noise. When ImVoxelNet meets 0.5-degree noise, its $AP_{3D}$ for easy samples decreases to 29.3%; meanwhile, our method maintains a performance of 35.7%. Additionally, our method attains the highest BEV accuracy, achieving a value of $AP_{BEV}$ of 55.3% for easy samples, thereby illustrating its effectiveness in utilizing C-V2X-transmitted vehicle positions for enhanced BEV perception.

In terms of calibration-free methods, CBR [10] delivers relatively lower performance, achieving $AP_{3D}$ scores of 24.7%, 15.7%, and 14.7% for easy, moderate, and hard samples, respectively. Notably, the performance of CBR drops significantly as the detection scenario becomes more challenging: the accuracy decreases by 9.0% from easy to moderate and by 10.0% from easy to hard. This indicates that CBR is less robust in complex environments, particularly when facing occlusions or limited visibility, which are common in real-world roadside deployments. In contrast, our proposed method achieves $AP_{3D}$ scores of 35.7%, 30.1%, and 28.6% under the same difficulty levels. The relative drops by 5.6% from easy to moderate and 7.1% from easy to hard, suggesting better robustness and spatial generalization. This improvement stems from the use of deformable cross-attention guided by vehicle positions and the injection of coordinate noise during training, which enable our model to adapt to uncertainty and partial occlusion more effectively.

#### 4.3.2. Ablation Studies

The results of the ablation study on the DAIR-V2X dataset, which evaluates the influence of vehicle position availability on BEV perception, are presented in Table 2. The results demonstrate that a higher proportion of vehicles transmitting positions through C-V2X significantly improves detection accuracy. Without the presence of position information (0%), the model attains a $AP_{3D}$ of 20.6% for easy samples and 15.7% for hard samples. When vehicle positions are accessible from a minimum of 25% of vehicles, performance significantly increases, achieving 35.7% for easy samples and 28.6% for hard samples. Similarly, $AP_{BEV}$ rises from 37.7% to 55.3% for easy samples, highlighting the essential contribution of position information in enhancing spatial alignment.

The model demonstrates an excellent accuracy level in the absence of vehicle position input, indicating its capacity to learn implicit spatial correspondences between roadside images and BEV features. This demonstrates the effectiveness of our method in managing situations with different levels of vehicle position availability.

To evaluate the model’s robustness and generalization under coordinate noise, we conducted a series of controlled experiments comparing different training inference noise settings. Table 3 summarizes the 3D and BEV detection results under the three following groups:

- Group A: No noise in training or inference, simulating an ideal condition.
- Group B: Trained without noise but tested with noise, simulating models without robustness training.
- Group C: Our proposed setting—noise added during both training and inference.

From Table 3, it can be seen that although Group A achieves the best results when inference is noise-free, its performance degrades significantly in the presence of inference noise. In contrast, our proposed strategy achieves slightly lower but stable performance across all difficulty levels. This confirms that injecting coordinate noise during training significantly improves the model’s generalization ability and robustness.

#### 4.3.3. Error Analysis of 3D Detection

**Figure 4:**
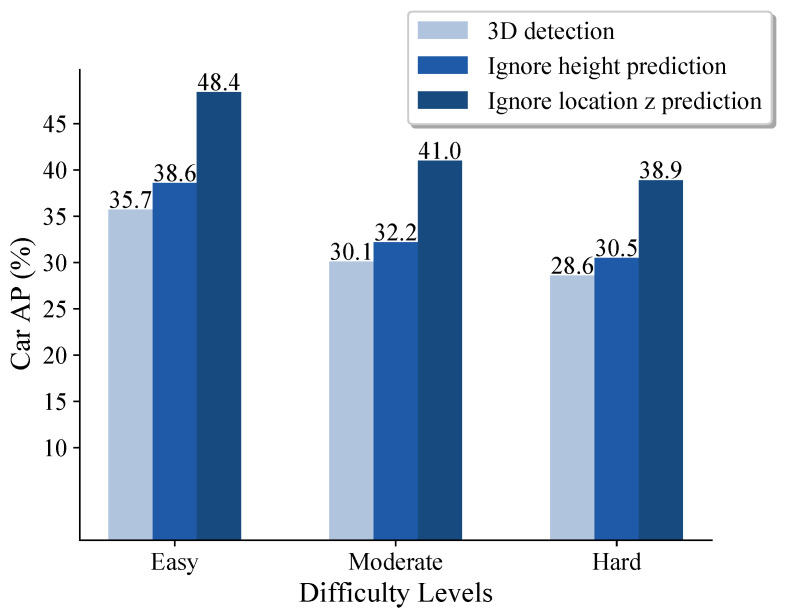
Error analysis of 3D detection under different difficulty levels.

To investigate potential reasons for the discrepancy between BEV detection results and 3D detection performance, we conducted an ablation study by separately ignoring height prediction and location z prediction in the 3D detection process. Figure 4 presents the corresponding results under different difficulty levels. The results show that ignoring height prediction leads to a moderate improvement, with $AP_{3D}$ increasing from 35.7%, 30.1%, and 28.6% to 38.6%, 32.2%, and 30.5% across easy, moderate, and hard difficulty levels, respectively. This suggests that height estimation introduces some uncertainty but is not the primary factor affecting 3D detection accuracy. In contrast, ignoring location z prediction results in a significant performance boost, achieving 48.4%, 41.0%, and 38.9% in easy, moderate, and hard difficulty levels. This substantial improvement indicates that predicting the z-axis location is particularly challenging in 3D detection, likely due to the varying installation heights of roadside cameras. Unlike vehicle-mounted sensors, which maintain a relatively fixed height, infrastructure-side cameras are often mounted at different elevations, leading to inconsistencies in depth estimation and increased difficulty in accurately localizing objects along the z-axis.

#### 4.3.4. Comparison on DAIR-V2X-I Dataset

In Table 4, we follow the comparison protocol used in the CBR paper [10]. We retain the same set of methods to ensure fair benchmarking on the DAIR-V2X-I dataset. Calibration-based methods like ImVoxelNet and BEVFormer depend significantly on precise camera parameters, attaining $AP_{3D}$ values of 44.8% and 61.4% for easy samples, respectively. Advanced methods such as BEVDepth and BEVHeight enhance detection accuracy to 75.5% and 77.8%, respectively.

Our method, utilizing DAIR-V2X pre-trained weights and fine-tuned over 200 epochs, attains 81.2% $AP_{3D}$ for easy samples and 80.3% for hard samples, exceeding all baseline performances. This indicates that utilizing pre-training on DAIR-V2X significantly improves feature learning and facilitates strong BEV perception independent of accurate camera calibration.

#### 4.3.5. Qualitative Analysis

**Figure 5:**
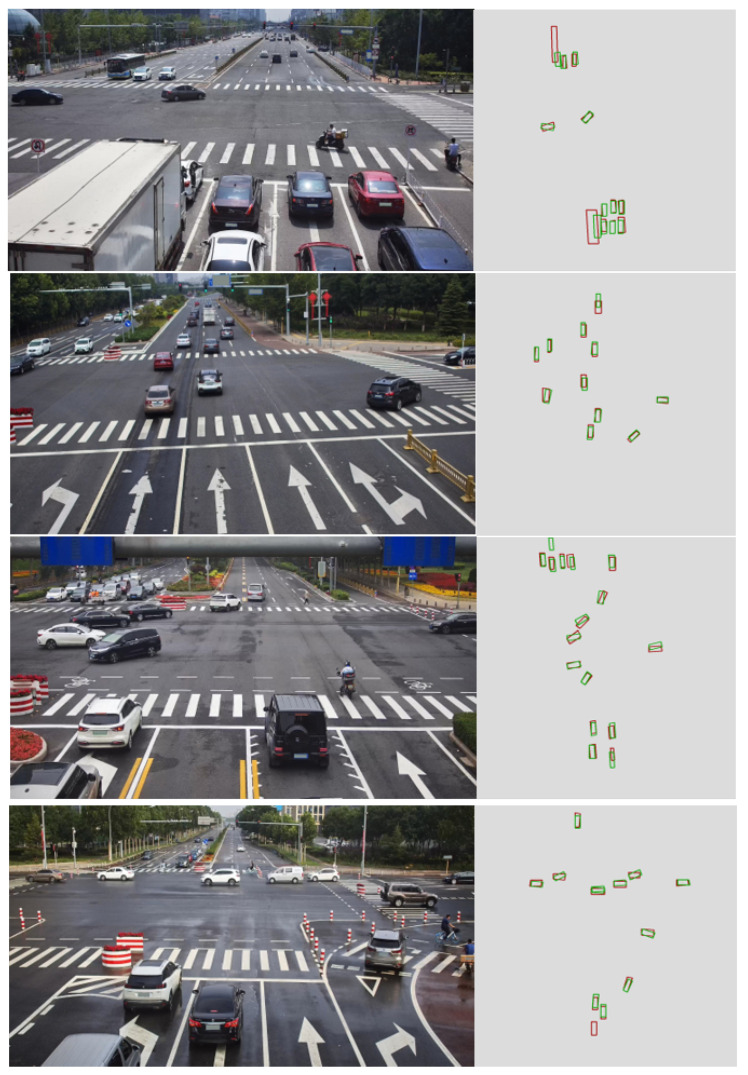
Visualization of detection results on the DAIR-V2X dataset. Ground truth boxes are shown in red and predicted results are shown in green. The method effectively detects vehicle dimensions and orientations, illustrating its robustness and applicability to cooperative driving scenarios.

Figure 5 illustrates the detection results on the DAIR-V2X dataset. The red boxes represent the ground truth, while the green boxes represent our predicted results. As shown in the figure, our proposed model is capable of accurately detecting vehicle locations and orientations even under noisy coordinate input. This visual evidence supports the quantitative findings presented earlier. In scenes with partial occlusion or vehicles located near the edge of the field of view, the model maintains correct alignment between vehicle positions and visual features, aided by the deformable attention mechanism. Furthermore, the bounding boxes of vehicles are well aligned with the ground truth in both size and orientation, demonstrating the robustness of our calibration-free approach in practical C-V2X scenarios. These results affirm the model is suitable for real-world applications.

#### 4.3.6. Deployment Performance

To evaluate the practical deployability of our proposed method, we measured its computational performance on the NVIDIA RTX 4090 GPU. The key deployment metrics are summarized in Table 5. The model has a parameter size of 35.445 M, and training on the DAIR-V2X dataset for 600 epochs took approximately 70 h with two GPUs. During inference on one GPU, the model achieves 40 frames per second (FPS), demonstrating its capability to operate in real-time scenarios.

Although our framework incorporates multi-scale deformable attention and transformer-based decoding, it is designed to be relatively lightweight. We adopted a ResNet-50 backbone and set both encoder and decoder depths to only three layers. This compact configuration effectively reduces the computational complexity compared to deeper transformer-based architectures. Furthermore, deformable attention modules are hardware-friendly due to their sparse sampling design, which limits the number of key–value operations and memory overhead. These characteristics make our approach feasible for real-time deployment in intelligent roadside units with modern edge computing capabilities.

## 5. Conclusions

In this paper, we propose a novel calibration-free framework for infrastructure-side BEV perception, which leverages vehicle positioning data transmitted via C-V2X communication to avoid reliance on camera calibration. This design addresses a key challenge in practical deployment, where intrinsic and extrinsic parameters are often difficult to obtain or unreliable due to environmental dynamics. To ensure robustness against potential localization errors from C-V2X noise and packet loss, we conducted experiments by injecting coordinate perturbations and varying the proportion of vehicles providing position information. The experimental results on the DAIR-V2X dataset demonstrate that our method surpasses both calibration-based and calibration-free baselines, achieving $AP_{3D}$ scores of 35.7%, 30.1%, and 28.6% for easy, moderate, and hard scenarios, respectively. On the DAIR-V2X-I dataset, our method achieves a competitive 81.2% $AP_{3D}$ on easy samples, benefiting from reduced occlusion and a broader field of view. Furthermore, our 3D detection error analysis reveals that predicting object positions along the z-axis remains a major challenge due to the varying installation heights of roadside cameras. This highlights the need for future improvements in depth-aware modeling under infrastructure constraints.

The proposed framework has broad applicability within ITSs. For instance, it can be deployed at urban intersections to assist in detecting pedestrians and vehicles obscured by large occluding objects, enhancing safety in complex environments. Furthermore, the system can support cooperative driving by broadcasting detected targets to approaching vehicles, enabling early reaction to sudden maneuvers. In addition, infrastructure-side perception may be used for real-time traffic event detection (e.g., stalled vehicles, unauthorized crossings), and this information can be disseminated to nearby agents for dynamic path planning. These use cases highlight the method’s capacity to enhance both individual vehicle perception and collective traffic intelligence.

While our framework focuses on algorithmic feasibility, future studies will investigate real-world system deployment. For example, in traffic accident warning scenarios, our system could integrate tracking algorithms to detect stationary vehicles, broadcasting alerts to surrounding vehicles for path planning. We will also consider realistic C-V2X communication problems, including transmit delays, collisions, and multipath propagation. In addition, we plan to address hardware deployment challenges, such as model compression for edge devices, end-to-end latency optimization to meet 100 ms response constraints, and camera layout optimization at intersections. Specifically, we will study the effect of camera height and spacing, as higher cameras offer better BEV projection but are constrained by practical installation limits. Further, we aim to explore multi-camera RSU fusion to cover large-scale intersections with minimal blind spots.

## Figures and Tables

**Table 1:** Performance comparison on the DAIR-V2X dataset. E., M., and H. denote easy, moderate, and hard difficulty levels, respectively.

Methods	Noise	Car ${\mathrm{AP}}_{3D}$@0.5			Car ${\mathrm{AP}}_{\mathrm{BEV}}$@0.5		
		E.	M.	H.	E.	M.	H.
ImVoxelNet [32]	/	47.6	29.2	27.1	51.9	32.7	30.4
	0.1	44.5	26.5	26.2	50.9	32.0	29.9
	0.2	38.6	23.1	22.6	45.1	26.8	26.4
	0.5	29.3	16.9	15.4	35.0	20.1	19.7
	1.0	19.7	11.4	10.2	25.5	14.7	14.3
CBR [10]	calibration-free	24.7	15.7	14.7	40.0	24.9	24.5
Ours	calibration-free	35.7	30.1	28.6	55.3	47.3	45.3

**Table 2:** Ablation study results on the DAIR-V2X dataset. E., M., and H. denote Easy, Moderate, and Hard difficulty levels, respectively.

Proportion	Car ${\mathrm{AP}}_{3D}$@0.5			Car ${\mathrm{AP}}_{\mathrm{BEV}}$@0.5		
	E.	M.	H.	E.	M.	H.
0%	20.6	17.4	15.7	37.7	32.3	30.0
random in {25–100%}	35.7	30.1	28.6	55.3	47.3	45.3

**Table 3:** Generalization performance under different coordinate noise settings. Evaluation is based on $AP_{3D}$ and $AP_{BEV}$ for the car class at IoU = 0.5.

Group	Train Noise	Infer Noise	${\mathrm{AP}}_{3D}$			${\mathrm{AP}}_{\mathrm{BEV}}$		
			E.	M.	H.	E.	M.	H.
A	✗	✗	38.4	32.1	30.6	57.9	49.5	47.8
B	✗	✓	30.3	24.5	22.7	46.8	38.0	35.7
C (ours)	✓	✓	35.7	30.1	28.6	55.3	47.3	45.3

**Table 4:** Performance comparison on the DAIR-V2X-I dataset. E., M., and H. denote easy, moderate, and hard difficulty levels, respectively.

Methods	Car ${\mathrm{AP}}_{3D}$@0.5		
	E.	M.	H.
ImVoxelNet [32]	44.8	37.6	37.6
BEVFormer [5]	61.4	50.7	50.7
BEVDepth [16]	75.5	63.6	63.7
BEVHeight [17]	77.8	65.8	65.9
CBR [10]	72.0	60.1	60.1
Ours	81.2	80.4	80.3

**Table 5:** Deployment performance of our model on NVIDIA RTX 4090.

Metric	Value
Parameters	35.445 M
Training Time	70 h (600 epochs)
Inference Speed	40 FPS

## Data Availability

The data used in this study are from the publicly available DAIR-V2X and DAIR-V2X-I datasets. The DAIR-V2X dataset, which supports vehicle-to-everything (V2X) perception tasks, is available at: https://github.com/AIR-THU/DAIR-V2X (accessed on 19 May 2025). The DAIR-V2X-I dataset (infrastructure-side only), which is used for roadside monocular 3D detection and BEV perception, is available at: https://github.com/AIR-THU/DAIR-V2X (accessed on 19 May 2025).
